# 2-Ethyl­imidazolium terephthalate

**DOI:** 10.1107/S1600536811038578

**Published:** 2011-09-30

**Authors:** Run-Qiang Zhu, Chun-Hua Yu

**Affiliations:** a Ordered Matter Science Research Center, College of Chemistry and Chemical Engineering, Southeast University, Nanjing 211189, People’s Republic of China

## Abstract

The asymmetric unit of the title compound, C_5_H_9_N_2_^+^·C_8_H_5_O_4_^−^, consists of one protonated 2-ethyl­imidazolium cation and two half terephthalate anions. The anions and cations are linked through N—H⋯O hydrogen bonds while the anions are associated *via* O—H⋯O inter­actions, resulting in a layered structure. The ethyl group of the cation is disordered over twosites of occupancies 0.812 (14) and 0.188 (14). The hydroxy H atoms of the anions are equally disordered over two symmetry-related sites.

## Related literature

The title compound was synthesized as part of our search for ferroelectric materials. For general background to ferroelectric organic frameworks, see: Fu *et al.* (2009 ▶); Ye *et al.* (2006 ▶); Zhang *et al.* (2008 ▶, 2010 ▶). For related structures, see: Tian (2007 ▶); Qu (2007 ▶). 
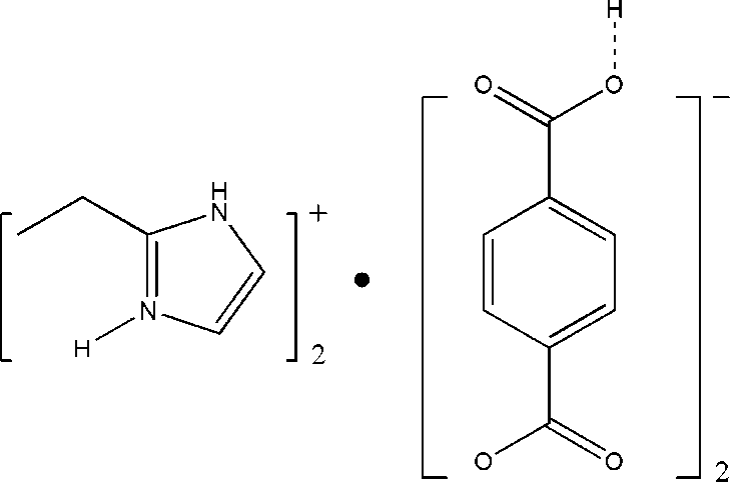


## Experimental

### 

#### Crystal data

C_5_H_9_N_2_^+^·C_8_H_5_O_4_^−^
*M* *_r_* = 262.26
Triclinic, 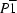

*a* = 6.4587 (13) Å
*b* = 7.0719 (14) Å
*c* = 14.688 (3) Å
α = 78.60 (3)°
β = 78.77 (3)°
γ = 89.50 (3)°
*V* = 644.7 (2) Å^3^
*Z* = 2
Mo *K*α radiation
μ = 0.10 mm^−1^
*T* = 293 K
0.30 × 0.25 × 0.20 mm

#### Data collection

Rigaku SCXmini diffractometer
Absorption correction: multi-scan (*CrystalClear*; Rigaku, 2005 ▶) *T*_min_ = 0.970, *T*_max_ = 0.980
6568 measured reflections
2933 independent reflections
2197 reflections with *I* > 2σ(*I*)
*R*_int_ = 0.034

#### Refinement

*R*[*F* ^2^ > 2σ(*F* ^2^)] = 0.060
*wR*(*F* ^2^) = 0.171
*S* = 1.07
2933 reflections
194 parameters
H-atom parameters constrained
Δρ_max_ = 0.29 e Å^−3^
Δρ_min_ = −0.24 e Å^−3^

### 

Data collection: *CrystalClear* (Rigaku, 2005 ▶); cell refinement: *CrystalClear*; data reduction: *CrystalClear*; program(s) used to solve structure: *SHELXS97* (Sheldrick, 2008 ▶); program(s) used to refine structure: *SHELXL97* (Sheldrick, 2008 ▶); molecular graphics: *DIAMOND* (Brandenburg & Putz, 2005 ▶); software used to prepare material for publication: *SHELXL97*.

## Supplementary Material

Crystal structure: contains datablock(s) I, global. DOI: 10.1107/S1600536811038578/vm2120sup1.cif

Structure factors: contains datablock(s) I. DOI: 10.1107/S1600536811038578/vm2120Isup2.hkl

Supplementary material file. DOI: 10.1107/S1600536811038578/vm2120Isup3.cml

Additional supplementary materials:  crystallographic information; 3D view; checkCIF report

## Figures and Tables

**Table 1:** Hydrogen-bond geometry (Å, °)

*D*—H⋯*A*	*D*—H	H⋯*A*	*D*⋯*A*	*D*—H⋯*A*
O4—H4*A*⋯O4^i^	0.82	1.67	2.452 (3)	160
O2—H2⋯O2^ii^	0.82	1.64	2.450 (3)	170
N2—H2*C*⋯O1^iii^	0.86	1.90	2.739 (3)	166
N1—H1*D*⋯O3	0.86	1.86	2.713 (3)	173

Symmetry codes: (i) 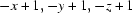; (ii) 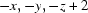; (iii) 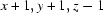.

**Fig. 1.**
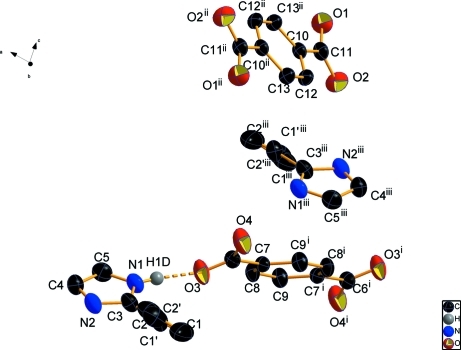
A partial packing diagram of the title compound, with displacement ellipsoids drawn at the 30% probability level. Only the major component of the disordered ethyl group is shown. Symmetry codes: (i) -x, -y, 1 - z; (ii) 1 - x, 1 - y, 2 - z; (iii) 2 - x, 1 - y, 1 - z.

**Fig. 2.**
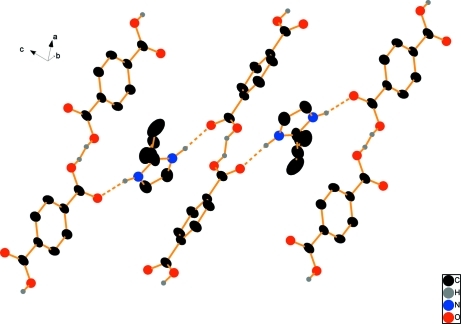
Packing diagram of the title compound, hydrogen bonds are shown as dashed lines. O···O interactions are shown as solid lines. Only the major component of the disordered ethyl group is shown.

## supplementary crystallographic information

### Comment

With reference to the compounds 2(C_4_H_7_N_2_)^+^.C_8_H_4_O_4_^2-^.2(C_4_H_6_N_2_).4(H_2_O) (Qu, 2007) and 2(C_3_H_5_N)^+^.C_8_H_4_O_4_^2- ^(Tian, 2007), we synthesized the title compound to find ferroelectric material by dielectric measurements of compound as a function of temperature (Fu *et al.*, 2009; Ye *et al.*, 2006; Zhang *et al.*, 2008; Zhang *et al.*, 2010). In the range from 190 K to near its melting point (m.p.>423 K), no dielectric anomaly was observed.

In the crystal structure (Fig. 1) determined at 293 K, we observe short O···O distances in the range 2.448 (3)–2.450 (3) Å between the interacting carboxylic acid groups. The H atoms attached to O2 and O4 are disordered. The O···O distances are much shorter than the N···O distances which are 2.714 (3) and 2.738 Å (Table 1).

### Experimental

A mixture of 2-ethyl imidazole (2.4 g, 25 mmol), terephthalic acid (25 mmol) in water was stirred for several days at ambient temperature. Colourless block crystals were obtained.

### Refinement

All H atoms were positioned geometrically and refined using a riding model, with C–H distances of 0.93–0.97Å, O—H = 0.82

Å and N–H = 0.86 Å. All H atoms were refined with isotropic displacement parameters set to 1.2 times the *U*_eq _of the parent atom, except for methyl and hydroxyl H atoms where it was set to 1.5 times the *U*_eq_ of the parent atom.

### Crystal data

**Table:**

C_5_H_9_N_2_^+^·C_8_H_5_O_4_^−^	*Z* = 2
*M**_r_* = 262.26	*F*(000) = 276
Triclinic, *P*1	*D*_x_ = 1.348 Mg m^−^^3^
Hall symbol: -P 1	Mo *K*α radiation, λ = 0.71073 Å
*a* = 6.4587 (13) Å	Cell parameters from 2933 reflections
*b* = 7.0719 (14) Å	θ = 2.2–27.5°
*c* = 14.688 (3) Å	µ = 0.10 mm^−^^1^
α = 78.60 (3)°	*T* = 293 K
β = 78.77 (3)°	Block, colourless
γ = 89.50 (3)°	0.30 × 0.25 × 0.20 mm
*V* = 644.7 (2) Å^3^	

### Data collection

**Table:**

Rigaku SCXmini diffractometer	2933 independent reflections
Radiation source: fine-focus sealed tube	2197 reflections with *I* > 2σ(*I*)
graphite	*R*_int_ = 0.034
CCD Profile fitting scans	θ_max_ = 27.5°, θ_min_ = 3.0°
Absorption correction: multi-scan (*CrystalClear*; Rigaku, 2005)	*h* = −8→8
*T*_min_ = 0.970, *T*_max_ = 0.980	*k* = −9→9
6568 measured reflections	*l* = −18→19

### Refinement

**Table:**

Refinement on *F*^2^	Secondary atom site location: difference Fourier map
Least-squares matrix: full	Hydrogen site location: inferred from neighbouring sites
*R*[*F*^2^ > 2σ(*F*^2^)] = 0.060	H-atom parameters constrained
*wR*(*F*^2^) = 0.171	*w* = 1/[σ^2^(*F*_o_^2^) + (0.0708*P*)^2^ + 0.3515*P*] where *P* = (*F*_o_^2^ + 2*F*_c_^2^)/3
*S* = 1.07	(Δ/σ)_max_ < 0.001
2933 reflections	Δρ_max_ = 0.29 e Å^−^^3^
194 parameters	Δρ_min_ = −0.24 e Å^−^^3^
0 restraints	Extinction correction: *SHELXL*, Fc^*^=kFc[1+0.001xFc^2^λ^3^/sin(2θ)]^-1/4^
Primary atom site location: structure-invariant direct methods	Extinction coefficient: 0.084 (10)

### Special details

**Table:**

Geometry. All e.s.d.'s (except the e.s.d. in the dihedral angle between two l.s. planes) are estimated using the full covariance matrix. The cell e.s.d.'s are taken into account individually in the estimation of e.s.d.'s in distances, angles and torsion angles; correlations between e.s.d.'s in cell parameters are only used when they are defined by crystal symmetry. An approximate (isotropic) treatment of cell e.s.d.'s is used for estimating e.s.d.'s involving l.s. planes.
Refinement. Refinement of *F*^2^ against ALL reflections. The weighted *R*-factor *wR* and goodness of fit *S* are based on *F*^2^, conventional *R*-factors *R* are based on *F*, with *F* set to zero for negative *F*^2^. The threshold expression of *F*^2^ > σ(*F*^2^) is used only for calculating *R*-factors(gt) *etc*. and is not relevant to the choice of reflections for refinement. *R*-factors based on *F*^2^ are statistically about twice as large as those based on *F*, and *R*- factors based on ALL data will be even larger.

### Fractional atomic coordinates and isotropic or equivalent isotropic displacement parameters (Å^2^)

**Table:**

	*x*	*y*	*z*	*U*_iso_*/*U*_eq_	Occ. (<1)
C1	0.7347 (7)	0.9795 (9)	0.2520 (3)	0.0599 (13)	0.808 (14)
H1A	0.6126	1.0576	0.2508	0.090*	0.808 (14)
H1B	0.8455	1.0340	0.1999	0.090*	0.808 (14)
H1C	0.7815	0.9756	0.3105	0.090*	0.808 (14)
C2	0.6784 (7)	0.7753 (6)	0.2440 (3)	0.0508 (13)	0.808 (14)
H2A	0.5645	0.7218	0.2961	0.061*	0.808 (14)
H2B	0.6286	0.7802	0.1854	0.061*	0.808 (14)
C1'	0.544 (5)	0.812 (5)	0.2476 (14)	0.113 (11)	0.192 (14)
H1'A	0.4549	0.9202	0.2539	0.170*	0.192 (14)
H1'B	0.4893	0.7036	0.2965	0.170*	0.192 (14)
H1'C	0.5486	0.7805	0.1867	0.170*	0.192 (14)
C2'	0.759 (4)	0.863 (4)	0.2564 (12)	0.068 (7)	0.192 (14)
H2'A	0.7635	0.8948	0.3174	0.082*	0.192 (14)
H2'B	0.8256	0.9671	0.2057	0.082*	0.192 (14)
C3	0.8617 (4)	0.6470 (4)	0.24548 (17)	0.0477 (6)	
C4	1.1731 (4)	0.5205 (4)	0.21494 (18)	0.0477 (6)	
H4	1.3152	0.5068	0.1905	0.057*	
C5	1.0399 (4)	0.3850 (4)	0.27270 (18)	0.0511 (6)	
H5	1.0717	0.2583	0.2960	0.061*	
C6	0.3529 (3)	0.2725 (3)	0.46010 (16)	0.0378 (5)	
C7	0.1695 (3)	0.1309 (3)	0.48246 (14)	0.0327 (5)	
C8	0.1917 (3)	−0.0380 (3)	0.44744 (16)	0.0392 (5)	
H8	0.3206	−0.0636	0.4118	0.047*	
C9	0.0230 (4)	−0.1691 (3)	0.46529 (16)	0.0398 (5)	
H9	0.0394	−0.2826	0.4421	0.048*	
C10	0.2463 (3)	0.1473 (3)	1.03616 (15)	0.0356 (5)	
C11	0.3795 (3)	0.3302 (3)	1.01537 (14)	0.0303 (5)	
C12	0.3127 (3)	0.5022 (3)	0.96744 (15)	0.0336 (5)	
H12	0.1871	0.5039	0.9453	0.040*	
C13	0.4326 (3)	0.6717 (3)	0.95247 (15)	0.0336 (5)	
H13	0.3867	0.7866	0.9208	0.040*	
N1	0.8498 (3)	0.4672 (3)	0.29095 (14)	0.0505 (6)	
H1D	0.7379	0.4096	0.3271	0.061*	
N2	1.0596 (3)	0.6826 (3)	0.19901 (14)	0.0472 (5)	
H2C	1.1087	0.7915	0.1642	0.057*	
O1	0.2779 (3)	0.0121 (2)	1.09758 (13)	0.0556 (5)	
O2	0.1042 (3)	0.1504 (2)	0.98639 (14)	0.0573 (5)	
H2	0.0476	0.0429	0.9968	0.086*	0.50
O3	0.4954 (3)	0.2675 (3)	0.39276 (13)	0.0595 (6)	
O4	0.3451 (3)	0.3907 (3)	0.51614 (14)	0.0648 (6)	
H4A	0.4374	0.4752	0.4941	0.097*	0.50

### Atomic displacement parameters (Å^2^)

**Table:**

	*U*^11^	*U*^22^	*U*^33^	*U*^12^	*U*^13^	*U*^23^
C1	0.082 (3)	0.044 (3)	0.053 (2)	0.011 (2)	−0.0086 (18)	−0.0121 (18)
C2	0.043 (3)	0.055 (2)	0.050 (2)	−0.0016 (17)	0.0018 (16)	−0.0096 (16)
C1'	0.09 (2)	0.21 (3)	0.051 (11)	0.055 (18)	−0.021 (11)	−0.040 (14)
C2'	0.088 (14)	0.072 (19)	0.044 (8)	−0.025 (13)	−0.010 (8)	−0.012 (9)
C3	0.0503 (14)	0.0481 (13)	0.0376 (12)	−0.0027 (11)	0.0033 (10)	−0.0032 (10)
C4	0.0409 (13)	0.0482 (14)	0.0496 (14)	−0.0041 (10)	−0.0002 (11)	−0.0078 (11)
C5	0.0583 (16)	0.0408 (13)	0.0500 (14)	−0.0033 (11)	−0.0084 (12)	−0.0010 (11)
C6	0.0347 (11)	0.0369 (11)	0.0402 (12)	−0.0150 (9)	−0.0043 (9)	−0.0063 (9)
C7	0.0297 (10)	0.0330 (10)	0.0338 (10)	−0.0121 (8)	−0.0050 (8)	−0.0037 (8)
C8	0.0307 (11)	0.0415 (12)	0.0442 (12)	−0.0085 (9)	0.0012 (9)	−0.0138 (9)
C9	0.0386 (12)	0.0334 (11)	0.0471 (13)	−0.0105 (9)	−0.0011 (10)	−0.0143 (9)
C10	0.0339 (11)	0.0308 (10)	0.0400 (11)	−0.0143 (8)	−0.0007 (9)	−0.0073 (9)
C11	0.0296 (10)	0.0272 (9)	0.0319 (10)	−0.0110 (8)	−0.0002 (8)	−0.0062 (8)
C12	0.0284 (10)	0.0341 (10)	0.0394 (11)	−0.0086 (8)	−0.0093 (8)	−0.0069 (8)
C13	0.0337 (11)	0.0264 (9)	0.0392 (11)	−0.0069 (8)	−0.0082 (9)	−0.0016 (8)
N1	0.0473 (12)	0.0518 (12)	0.0420 (11)	−0.0162 (9)	0.0033 (9)	0.0040 (9)
N2	0.0486 (12)	0.0369 (10)	0.0459 (11)	−0.0128 (9)	0.0060 (9)	0.0019 (8)
O1	0.0599 (11)	0.0355 (9)	0.0645 (12)	−0.0245 (8)	−0.0137 (9)	0.0091 (8)
O2	0.0574 (11)	0.0468 (10)	0.0699 (12)	−0.0323 (9)	−0.0257 (10)	−0.0024 (9)
O3	0.0464 (10)	0.0627 (12)	0.0623 (12)	−0.0298 (9)	0.0151 (9)	−0.0197 (9)
O4	0.0571 (12)	0.0692 (13)	0.0679 (13)	−0.0384 (10)	0.0113 (9)	−0.0345 (10)

### Geometric parameters (Å, °)

**Table:**

C1—C2	1.525 (7)	C6—O4	1.278 (3)
C1—H1A	0.9600	C6—C7	1.501 (3)
C1—H1B	0.9600	C7—C9^i^	1.383 (3)
C1—H1C	0.9600	C7—C8	1.387 (3)
C2—C3	1.486 (5)	C8—C9	1.388 (3)
C2—H2A	0.9700	C8—H8	0.9300
C2—H2B	0.9700	C9—C7^i^	1.383 (3)
C1'—C2'	1.47 (4)	C9—H9	0.9300
C1'—H1'A	0.9600	C10—O1	1.224 (3)
C1'—H1'B	0.9600	C10—O2	1.277 (3)
C1'—H1'C	0.9600	C10—C11	1.506 (3)
C2'—C3	1.69 (3)	C11—C13^ii^	1.384 (3)
C2'—H2'A	0.9700	C11—C12	1.390 (3)
C2'—H2'B	0.9700	C12—C13	1.389 (3)
C3—N1	1.310 (3)	C12—H12	0.9300
C3—N2	1.327 (3)	C13—C11^ii^	1.384 (3)
C4—C5	1.337 (3)	C13—H13	0.9300
C4—N2	1.361 (3)	N1—H1D	0.8600
C4—H4	0.9300	N2—H2C	0.8600
C5—N1	1.356 (4)	O2—H2	0.8200
C5—H5	0.9300	O4—H4A	0.8200
C6—O3	1.219 (3)		
			
C2—C1—H1A	109.5	C4—C5—H5	126.5
C2—C1—H1B	109.5	N1—C5—H5	126.5
H1A—C1—H1B	109.5	O3—C6—O4	125.1 (2)
C2—C1—H1C	109.5	O3—C6—C7	119.7 (2)
H1A—C1—H1C	109.5	O4—C6—C7	115.20 (19)
H1B—C1—H1C	109.5	C9^i^—C7—C8	119.54 (18)
C3—C2—C1	112.1 (4)	C9^i^—C7—C6	121.07 (19)
C3—C2—H2A	109.2	C8—C7—C6	119.35 (19)
C1—C2—H2A	109.2	C7—C8—C9	120.4 (2)
C3—C2—H2B	109.2	C7—C8—H8	119.8
C1—C2—H2B	109.2	C9—C8—H8	119.8
H2A—C2—H2B	107.9	C7^i^—C9—C8	120.0 (2)
C2'—C1'—H1'A	109.5	C7^i^—C9—H9	120.0
C2'—C1'—H1'B	109.5	C8—C9—H9	120.0
H1'A—C1'—H1'B	109.5	O1—C10—O2	125.55 (19)
C2'—C1'—H1'C	109.5	O1—C10—C11	119.7 (2)
H1'A—C1'—H1'C	109.5	O2—C10—C11	114.72 (19)
H1'B—C1'—H1'C	109.5	C13^ii^—C11—C12	119.61 (18)
C1'—C2'—C3	96 (2)	C13^ii^—C11—C10	119.44 (18)
C1'—C2'—H2'A	112.6	C12—C11—C10	120.91 (18)
C3—C2'—H2'A	112.6	C13—C12—C11	120.29 (19)
C1'—C2'—H2'B	112.6	C13—C12—H12	119.9
C3—C2'—H2'B	112.6	C11—C12—H12	119.9
H2'A—C2'—H2'B	110.1	C11^ii^—C13—C12	120.10 (19)
N1—C3—N2	107.0 (2)	C11^ii^—C13—H13	120.0
N1—C3—C2	124.2 (3)	C12—C13—H13	120.0
N2—C3—C2	128.7 (2)	C3—N1—C5	110.0 (2)
N1—C3—C2'	140.7 (6)	C3—N1—H1D	125.0
N2—C3—C2'	106.2 (7)	C5—N1—H1D	125.0
C2—C3—C2'	31.6 (7)	C3—N2—C4	109.5 (2)
C5—C4—N2	106.4 (2)	C3—N2—H2C	125.2
C5—C4—H4	126.8	C4—N2—H2C	125.2
N2—C4—H4	126.8	C10—O2—H2	109.5
C4—C5—N1	107.0 (2)	C6—O4—H4A	109.5

Symmetry codes: (i) −*x*, −*y*, −*z*+1; (ii) −*x*+1, −*y*+1, −*z*+2.

### Hydrogen-bond geometry (Å, °)

**Table:**

*D*—H···*A*	*D*—H	H···*A*	*D*···*A*	*D*—H···*A*
O4—H4A···O4^iii^	0.82	1.67	2.452 (3)	160.
O2—H2···O2^iv^	0.82	1.64	2.450 (3)	170.
N2—H2C···O1^v^	0.86	1.90	2.739 (3)	166.
N1—H1D···O3	0.86	1.86	2.713 (3)	173.

Symmetry codes: (iii) −*x*+1, −*y*+1, −*z*+1; (iv) −*x*, −*y*, −*z*+2; (v) *x*+1, *y*+1, *z*−1.
